# Cyclosporine: A Review

**DOI:** 10.1155/2012/230386

**Published:** 2012-01-04

**Authors:** Dustin Tedesco, Lukas Haragsim

**Affiliations:** Division of Nephrology and Hypertension, University of Oklahoma Health Sciences Center, 920 S. L. Young Blvd, WP 2250 Oklahoma City, OK 73104, USA

## Abstract

The discovery and use of cyclosporine since its inception into clinical use in the late 1970s has played a major role in the advancement of transplant medicine. While it has improved rates of acute rejection and early graft survival, data on long-term survival of renal allografts is less convincing. The finding of acute reversible nephrotoxicity and nephrotoxicity in nonrenal transplants has since led to the widely accepted view that there is a chronic more irreversible component to this agent as well. Since that time, there has been intense interest in finding protocols which seek to minimize and even avoid the use of calcineurin inhibitors altogether. We seek to review cyclosporine in terms of its mechanism of action, pathophysiologic, and histologic features associated with acute and chronic nephrotoxicity and recent studies looking to avoid its toxic side effects.

## 1. Introduction

Discovered in the lab of Sandoz in Switzerland in 1972, cyclosporine (CsA) has since revolutionized transplant medicine. Initially discovered while searching for novel antifungal agents, it was found to have many immunologic properties that made it an attractive agent for immunosuppression following renal and other solid organ transplants. With the premise that cell-mediated immunity was involved in autoimmune and chronic inflammatory conditions, Borel set up a series of experiments using antiinflammatory, immunosuppressant, and antimitotic medications to examine their effects on lymphocyte-mediated lysis of presensitized and naïve effector cells. In these experiments, it was found that cyclosporine inhibited both in vitro cell-mediated lysis as well as lymphocyte sensitization by allogeneic target cells [1]. It was this work and others by Borel that exhibited the cell-mediated specificity of cyclosporine, theoretically lending itself to a far better side effect profile than the current immunosuppressive agents in use at that time. Subsequently, a European multicenter trial demonstrated one-year graft survival of 72% and 52% in recipients of cadaveric renal transplants allocated to receive either cyclosporine or azathioprine and steroids, respectively, for immunosuppression. Such promising results helped lead to clinical approval of CsA for use in the early 1980s [2]. With improved rates of acute rejection and one-year graft survival, cyclosporine has become a mainstay for immune suppression of renal and other solid organ transplants. A review from Hariharan et al. published in 2000 looking at graft survival in more than 93,000 transplants from 1988 to 1996 revealed one-year graft survival rates of 94 and 88% in living related and deceased donor allografts, respectively [3]. The most recent data from United Network for Organ Sharing (UNOS) from 1998 to 2007 reveals current one-year adjusted survival rates to be 96.6 and 91.6% in living related and deceased donor renal allografts, respectively. Despite the marked improvement in the rates of acute rejection and one-year graft survival, long-term data has been somewhat disappointing, with current age-adjusted graft survival at five years only 81.4% in living related donors and 71.6% in those with deceased donor transplants. This difference has largely been attributed to the nephrotoxic effects of cyclosporine.

That cyclosporine is nephrotoxic was discovered even with its initial use. In the first attempt at using cyclosporine for immunosuppression following transplant, Calne et al. using a dose of 25 mg/kg found a significant but unexpected nephrotoxicity that was not seen in initial animal experiments [4]. Follow-up studies by the same group using a slightly lower dose (17 mg/kg) and selecting for primary functioning allografts showed improved outcomes with this new strategy with a one-year predicted graft survival rate of 86% [5, 6]. Subsequent studies in which renal allograft recipients who received CsA for 90 days were switched to conventional treatment with azathioprine, and corticosteroids demonstrated improvement in renal function similar to controls [7, 8]. Other studies have also demonstrated improved GFR and blood pressure measurements using a target of fifty percent of the standard area-under-the-curve (AUC) dose [9]. Thus, it appeared that CsA nephrotoxicity may be dose dependent and reversible upon dose reductions or discontinuation of the drug. Since that time, one of the major reasons given for the lack of long-term improvement in graft survival has been chronic calcineurin (CNI) nephrotoxicity. While this has been the source of much debate, the overall attitude has been one of acceptance. Taken together with its nephrotoxicity and other well-established nonimmunologic side effects, there is now strong interest in creating calcineurin-free protocols for the prevention of transplant rejection. This dates back historically, as early as the 1990s in which the desire to reduce CsA exposure without risking under immunosuppression led to protocols including two other immunosuppressive agents. Taken a step further was the use of antilymphocytic antibodies in the early posttransplant period in an attempt to avoid early CsA exposure altogether until such time that allograft function was fully recovered [10]. 

## 2. Structure and Clinical Pharmacology

Cyclosporine is a lipophilic, cyclic endecapeptide with a molecular weight of 1202 Daltons [11]. In plasma, it is 90% protein bound, mostly to lipoproteins, but also to albumin and globulins. In blood, cyclosporine is extensively distributed in erythrocytes. There are differences in bioavailability of cyclosporine in large part due to significant interindividual variability in intestinal absorption, a process that is further influenced by food ingestion, diabetes, gastric motility problems, and diarrhea among other things [12]. However, variability in intestinal absorption is an effect that has been dampened by the creation of the microemulsion formulations. Kovarik et al. demonstrated a more stable concentration-time profile and bioequivalent peak-trough fluctuation in both fasting daytime and nonfasting nighttime administration of the microemulsion formulation when compared to that of the commercially available counterpart. Furthermore, there was a 30% increase in AUC in the microemulsion formulation due primarily to absorption-related pharmacokinetic differences [13]. Metabolism is primarily hepatic with a half-life of 6.4–8.7 hours with less than 1% appearing in the urine or feces. After metabolism by the cytochrome P450 system, primarily CYP3A4, and CYP3A5, CsA metabolites are eliminated in the bile with less than 5% excreted in the urine. It is thought that variable expression of the isoenzymes, CYP3A4, and CYP3A5 plays a role in cyclosporine's unpredictable bioavailability [12, 14]. Coadministration of medications known to inhibit the cytochrome P-450 system (ketoconazole, erythromycin, calcium-channel blockers, and others) is known to increase cyclosporine levels. Likewise, inducers of the P-450 system, such as phenytoin, phenobarbital, carbamazepine, and valproate, decrease cyclosporine levels [11].

## 3. Mechanism of Action

Calcineurin is a calcium/calmodulin-dependent serine threonine protein phosphatase. Activated calcineurin dephosphorylates regulatory sites on several transcription factors, most notably nuclear factor of activated T-lymphocytes (NFATs). Inhibition of calcineurin by cyclosporine occurs via binding to the immunophilin, cyclophilin. It is this step that prevents the dephosphorylation of NFAT and its subsequent translocation from the cytoplasm to the nucleus in an IL-2-mediated process. Inhibition at this level thereby prevents activation of promoters of T-cell activation and overall immune response.

In addition to its effects on immune function, CsA possesses several other toxic effects. The most notable is acute and chronic nephrotoxicity, but also include hypertension, hyperlipidemia, gingival hyperplasia, hyperkalemia, neurotoxicity, hypomagnesaemia, hyperuricemia, and thrombotic microangiopathy [11]. These effects are thought in part due to calcineurin inhibition in nonlymphatic tissues [15]. The electrolyte disturbances are believed due to alterations in tubular function and thereby ion homeostasis [12, 16]. The nephrotoxic effects have garnered the most attention over the years and have two components, an acute nephrotoxicity caused by vascular dysfunction and a more chronic fibrotic form.

## 4. Pathophysiology of CNI Nephrotoxicity

### 4.1. Acute Nephrotoxicity

The findings of nephrotoxicity in early studies using CsA as an immunosuppressant led to much research into the pathophysiology of this process. Vasoconstriction of the afferent arterioles was first suggested by Murray et al. in 1985, in which conscious rats were administered CsA infusions (20 mg/kg) resulting in a significant reduction in renal blood flow and a rise in renal vascular resistance. This was proposed due to activation of the renal sympathetic nervous system as there was demonstration of a concomitant stimulation of plasma renin activity. Also noted was a reduction in the rate of decline of renal blood flow in denervated rats [17]. Similarly, Barros et al. demonstrated increase in vascular resistance in afferent and efferent arterioles with a reduction in renal plasma flow and GFR, an effect that was attenuated by pretreatment with the angiotensin-converting enzyme inhibitor captopril and the calcium channel blocker, verapamil [18]. This vascular mediated effect stems from an imbalance in vasoconstrictor and vasodilator factors. Cyclosporine has been shown to increase the vasoconstrictor factors endothelin as well as thromboxane in addition to its activation of the renin-angiotensin system (RAS). Also demonstrated is a reduction in the vasodilator factors, prostacyclin, prostaglandin E2, and NO [19, 20]. Activation of the RAS by CsA is by two mechanisms, a direct effect on juxtaglomerular cells (JG) and indirectly through arterial vasoconstriction and reduced renal plasma flow. The direct effect of CsA on JG cells was demonstrated in the late 1980s by Kurtz et al. In this study, primary cell cultures from rat renal cortex containing JG cells showed a threefold increase in renin secretion upon exposure to cyclosporine. Furthermore, no increase in prostaglandin formation or increase in cyclic AMP concentration was observed. This led to the conclusion that CsA-stimulated renin secretion by direct effects on JG cells [21]. Another interesting observation in the potential pathogenic mechanisms of vasoconstriction is that by Höcherl et al. who demonstrated that CsA markedly lowered COX-2 expression which has been shown to have binding sites for NFAT. Therefore, the inhibition of calcineurin by CsA leads to a reduction in NFAT-mediated COX-2 expression and downstream production of arachidonic acid metabolites thereby favoring vasoconstriction [22]. Other mechanisms have been proposed as well including the demonstration that CsA leads to mesangial cell contraction with subsequent alterations in glomerular permeability, endothelin dysfunction, production of oxygen-free radicals and superoxide, and interference with normal tubular function as previously mentioned [23].

The role of the innate immune system has also been implicated in the nephrotoxicity of CsA. Recent reports suggest that upregulation of toll-like receptors (TLR) and TNF-*α*, responsible for dendritic cell maturation, may be stimulated by endogenous, noninfectious ligands (i.e., injured tubular epithelial cells) and stimulates secretion of chemokines that initiate phagocytic influx and immune activation. A study done by Lim et al. demonstrated through RT-PCR upregulation of TLR2, TLR4, and TNF-*α* mRNA in CsA-treated rats. They also demonstrated increased levels of MHC-II by immunohistochemistry. Thus, it may be reasonable to conclude that activation of TLR2 and TLR4 by injured renal tubular cells caused by CsA provides a link between innate immunity and the direct toxic effects of CsA on renal tubular cells [24].

### 4.2. Chronic Nephrotoxicity

The most notably referenced study linking cyclosporine with chronic nephrotoxicity was that done in 1984 by Myers et al. in which recipients of cardiac transplants surviving greater than 12 months and treated with CsA were compared to a similar group transplanted prior to 1980 who received azathioprine and steroids. Data at one month and out to one year revealed significant reductions in GFR, renal plasma flow, and renal blood flow. Also, biopsy of five CsA-treated patients revealed tubulointerstitial injury and focal glomerular sclerosis, the intensity of which appeared to correlate with degree of renal impairment [25]. Further evidence for chronic nephrotoxicity related to long-term cyclosporine use is the clinical and pathologic findings of impaired renal function in heart, liver, and lung transplants as well as patients with autoimmune disease treated with cyclosporine [26–29].

## 5. Pathologic Findings

The hallmark finding in CNI nephrotoxicity is arteriolar hyalinosis, which is characterized by nodular hyaline deposits in the tunica media of afferent arterioles. Another commonly described finding is that of interstitial or so-called striped fibrosis. This is hypothesized to be secondary to the aforementioned vasoconstrictive effects of CsA with subsequent arteriolar luminal narrowing. The subsequent tissue ischemia/hypoxia leads to a reperfusion type injury with the formation of reactive oxygen species and free radicals leading to cellular injury and apoptosis [12, 23]. Cyclosporine has also been shown to upregulate TGF-*β* expression in juxtaglomerular cells. TGF-*β* is known to promote fibrosis through its increase in the production of extracellular matrix proteins and induction of epithelial mesenchymal transition [12, 30].

Activation of the RAS appears important for the development of CNI nephrotoxicity not only for its vasoconstrictive effects but also proinflammatory and profibrogenic effects. This is thought due to the action of angiotensin II which has been shown to induce fibrosis mostly through induction of TGF-*β* [12, 31, 32]. 

Perhaps, the most well-known study documenting the long-term nephrotoxic effects of cyclosporine and its associated pathologic findings is that by Nankivell et al. in 2004. The authors attempted to look at the histologic evolution of CsA nephrotoxicity by examining protocol kidney biopsies. Biopsy was performed at the time of implantation, again at weeks one, two, and four and then months three, six, and twelve, then yearly thereafter for ten years. In total, 888 study biopsies were obtained in 99 patients yielding eight biopsies per patient. At ten years, the point prevalence for lesions considered to be consistent with chronic cyclosporine toxicity was 100%. Importantly, other causes of hyalinosis were ruled out including donor hyalinosis, hypertension, ischemic injury, dyslipidemia, and hyperglycemia. The authors concluded from this work that long-term immunosuppression with CsA was inappropriate for renal transplant recipients and that strategies for avoidance of calcineurin inhibitors be validated [33]. While studies were already in progress looking at ways to avoid or minimize exposure of renal allograft recipients to the effects of CNI, the study by Nankivell seemed further validation.

## 6. Calcineurin-Inhibitor-Sparing Protocols

### 6.1. Calcineurin Inhibitor Avoidance

One of the earliest studies attempting to avoid CNI was that by Vincenti et al. in which 98 patients receiving either cadaveric or living donor kidneys received daclizumab (an IL-2 receptor blocker) plus mycophenolate mofetil (MMF) and corticosteroids. Results on primary efficacy revealed unacceptably high rates of biopsy-proven rejection (48% at 6 months) [34]. Additionally, Larson et al. found no difference at one year in either patient or allograft survival nor incidence of acute rejection using sirolimus along with MMF and corticosteroids [35].

### 6.2. Calcineurin Inhibitor Avoidance and/or Withdrawal

More recently, three large studies have been published examining this topic. In December 2007, the ELITE-SYMPHONY study examined 1645 renal transplants patients who were designated to receive either standard therapy with CsA, MMF, and corticosteroids or undergo daclizumab induction MMF and corticosteroids and either low-dose tacrolimus, low-dose CsA, or low-dose sirolimus. The primary endpoint was GFR at 12 months. Results showed that patients in the low-dose tacrolimus group had better GFR, highest graft survival, and lowest rate of biopsy-proven rejection. This was followed by the low-dose CsA group. The group receiving low-dose sirolimus had worse outcomes and more serious adverse events [36].

Similarly, the CAESAR study by Ekberg et al. used a regimen of daclizumab induction followed by MMF and corticosteroids and either CsA withdrawal by six months, low-dose CsA (trough 50–100 ng/mL) for 12 months, or standard CsA (target trough 150–300 ng/mL) for 12 months. Mean GFR at 12 months (primary end point) did not differ significantly among the three groups. However, rates of biopsy-proven rejection were significantly higher in the CsA withdrawal group [37]. Followup to the CAESAR study examining the pharmacokinetics of mycophenolic acid (MPA) in relation to cyclosporine revealed higher MPA AUC in the cyclosporine group as a result of enterohepatic recirculation. Despite this increase, it was suggested that dose adjustments be made in MMF in the absence of CsA to avoid underexposure and risk of acute rejection [38].

Finally, the BENEFIT study in 2010 examined the effects of the costimulation blocker belatecept versus cyclosporine in preservation of renal function in living or deceased donor renal transplants. Patients receiving either a more or less intense regimen of belatacept experienced better renal function and overall patient/graft survival but had higher incidence and grade of acute rejection [39].

Taken together, the above studies demonstrate that while attempts at minimizing or avoiding CNI altogether have resulted in similar patient and allograft survival, rates of acute rejection may be too great for the adoption of these protocols on a routine basis. Similarly, one should use caution in consideration of steroid-sparing protocols. While short and intermediate outcomes show equivalent patient and graft survival, long-term results may be less promising. The presence of interstitial fibrosis and tubular atrophy may in fact be greater in steroid-free patients leading to decreased long-term graft function. In addition, although discontinuation of steroid therapy early after transplant may reduce the risk of rejection, there are no studies evaluating different times in the first year at which steroid therapy can safely be discontinued [40].

 While the toxic side effect of acute nephrotoxicity as a result of cyclosporine administration has been well documented and widely accepted, the concept of chronic nephrotoxicity seems a matter still up for debate. Several issues have been raised lately by opponents of this idea. First, the histologic lesions thought characteristics of chronic CNI nephrotoxicity are nonspecific. The findings of arteriolar hyalinosis can also be seen with preexisting donor injury, age, hypertension, and diabetes, as can the findings of interstitial fibrosis, tubular atrophy, and glomerulosclerosis. The findings of tubular microcalcification can also be seen with preexisting donor injury, ischemic tubular injury, proteinuria, and acute tubular necrosis. In addition, many of the above findings can be seen with recurrence of the primary disease. Thus, when evaluating late deterioration in allograft function, one must rule out several other common causes before labeling it as chronic CNI toxicity [12]. Furthermore, several of the studies mentioned above evaluating CNI avoidance or sparing have failed to prove long-term benefit [41]. In fact, in the majority of the studies already mentioned in this review, the authors found either no difference in outcomes or found that those in the CNI avoidance group had the worst outcomes, usually in regards to rates of acute rejection. In a retrospective analysis of 1663 kidney transplant patients, no correlation was found with CsA levels and change in serum creatinine (SCr) or episodes of acute rejection (although higher rejection rates were seen with lower CsA doses at four to six months) over 36 month followup. Moreover, they found no evidence of progressive nephropathy with cyclosporine and concluded that graft loss was most commonly due to acute rejection and chronic graft dysfunction [42]. Looking again at the previously mentioned study by Nankivell et al., opponents point out that while histologic abnormalities developed, the 10-year death-censored graft survival rate was 95% and the mean SCr was 1.6 mg/dL [33]. Moreover, the same group has published a report in which MMF was used in place of azathioprine, along with CsA and corticosteroids. Findings demonstrated less interstitial fibrosis, striped fibrosis, glomerulosclerosis, and mesangial matrix accumulation, lesions previously attributed to long-term use of CNI. They also reported decreased rates of acute rejection and delayed expression of CsA nephrotoxicity [41, 43]. Overall, this suggests factors other than cyclosporine alone contribute to chronic allograft dysfunction. Additional findings calling into question the significance of chronic calcineurin exposure to late graft failure are those of the DeKAF study. Results of this study in which one hundred seventy-three renal transplant patients with late graft failure underwent biopsy showed that evidence of antibody-mediated rejection was common and that risk of graft loss was greatest in those with C4d+ staining. Diagnosis of calcineurin inhibitor nephrotoxicity did not impact the risk of late graft failure in this study [44].

What about data on renal function in nonrenal transplants? As mentioned above, Myers et al. demonstrated reduced GFR, renal plasma flow, and renal blood flow in native kidneys of cardiac transplant recipients. Opponents are quick to point out that the average dose of CsA was quite high at 17.5 mg/day with trough levels ranging from 300 to 350 ng/dL for the first four months following transplant and were still 164 ng/mL out to two years [25, 41].

More recently, Ojo et al. reviewed the incidence of chronic renal failure and associated risk factors in nonrenal transplant patients in the US from 1990 to 2000. Results indicated the greatest risk for development of chronic renal failure were increasing age, female sex, pretransplant hepatitis C, hypertension, diabetes, and postoperative renal failure. Use of CNI was not found to be a significant cause [45]. Similarly, previous reports examining early surveillance biopsies for predictors of renal allograft dysfunction have shown that biopsies with active inflammation have worse graft function after one year. Importantly, in these studies, there was no correlation between use of CNI or CNI levels and allograft function [46–48]. The above suggests that low-level alloreactivity with subclinical rejection is likely another factor in late allograft dysfunction.

Finally, if cyclosporine (and tacrolimus) is responsible for chronic nephrotoxicity, why are some patients seemingly immune to its effects? As mentioned earlier, there is large interindividual variability in many of the pharmacokinetic properties of cyclosporine. However, studies do exist in nonrenal transplants where CsA exposure has been minimized or CsA withdrawn altogether using replacement with MMF or a combination of Everolimus and low-dose CsA showing noninferiority with respect to renal function and rates of acute rejection [49]. Furthermore, a study by Dharancy et al. demonstrated a significant increase in eGFR in liver transplant patients switched from a CNI-containing regimen to MMF monotherapy five years after liver transplant with correspondingly low rates of rejection suggesting CNI avoidance can and possibly should be considered [50].

More specific in regards to the aforementioned interindividual variability in pharmacokinetics is the role of drug transporters and drug metabolizing enzymes. Hesselink et al. recently reviewed the role of the drug transporter adenosine triphosphate-binding cassette protein B1 (ABCB1) and the enzymes of the cytochrome P450 system, specifically CYP3A4 and CYP3A5.

ABCB1 is a 170 kDa ATP-dependent drug transporter responsible for transporting drugs from the cytoplasm to the cell surface and ultimately into the extracellular space. In human kidneys, ABCB1 is found most prominently in the brush border of proximal tubular epithelial cells. ABCB1 has been shown to be upregulated in the setting of CsA exposure, which likely serves as a protective mechanism against CsA exposure. Likewise, demonstration of lower ABCB1 expression has been shown to be a risk factor for development of chronic histologic changes in CNI-treated renal allograft recipients [14, 51].

CYP3A4 and to a larger extent CYP3A5 are the major isoenzymes responsible for the hepatic metabolism of cyclosporine. Studies have shown reduced intrarenal expression of CYP3A5 in renal biopsies which may be a risk factor for nephrotoxicity in patients treated with CNI [14, 52]. Genetic associations examining the effects of single nucleotide polymorphisms (SNPs) suggest little role in regards to CNI pharmacokinetics. Renal transplant patients carrying the CYP3A5*3 allele appear to require lower doses of CNI to achieve target concentrations [53]. In regards to ABCB1, studies have demonstrated that donor genotype ABCB1 3435 TT is associated with CNI nephrotoxicity and higher grades of IF/TA [44, 54]. Conversely, other studies failed to demonstrate a correlation between allograft survival and ABCB 1 genotype [55]. Even fewer data are available for the role of genetic variation in CYP3A and CNI-associated renal dysfunction. One study suggested biopsy-proven tacrolimus nephrotoxicity in renal allograft recipients with CYP3A4*1/CYP3A5*1 and CYP3A4*1B/YP3A5*1 genotypes [56]. Conversely, subsequent studies have found no significant association with CYP3A5 genotype and CNI-mediated nephrotoxicity [57]. Overall, data on this subject are conflicting. Importantly, to date, there is no direct evidence of higher intrarenal CNI concentrations as a result of certain ABCB1 expression/genotype leading to CNI nephrotoxicity [14]. Further study in this area is warranted.

With large interindividual variability in CsA pharmacokinetics, the known difference in allograft function and subsequent survival with respect to living versus deceased donor donation, and the availability of a greater number of immunosuppression agents, it is clear that immunosuppressive treatment should be individualized to each patient, even if this means the possibility of CNI avoidance.

## 7. Summary

Cyclosporine has no doubt revolutionized transplant medicine since its first clinical use in the late 1970s, improving rates of acute rejection and early graft survival. Despite the early discovery of acute, and more recently suggested chronic nephrotoxicity, the use of CNI continues to be a mainstay in transplant medicine. While the latter concept is still being actively debated, the nonimmunologic side effects of the calcineurin inhibitors cyclosporine and tacrolimus warrant continued research into effective protocols achieving fewer side effects while maintaining low risk of rejection. While data in this regard are promising, none appears so overwhelming so as to supplant CNI as a treatment for the prevention of transplant rejection. 
